# Intraoperative use of the EndoScell scanner to assist in the surgical resection of papillary thyroid carcinoma: A case report

**DOI:** 10.1097/MD.0000000000048433

**Published:** 2026-04-24

**Authors:** Yaqian Liu, Zhuoyang Zhou, Lixiao Ma, Zeyu Zhang, Fenghua Zhang

**Affiliations:** a Department of Clinical Medicine and Surgery, Hebei Medical University, Shijiazhuang, China; b Department of Clinical Medicine and Surgery, North China University of Science and Technology, Tangshan, China; c Department of Clinical Medicine and Surgery, Hebei North University, Zhangjiakou, China; d Department of Gland Surgery, Hebei Provincial People’s Hospital, Shijiazhuang, China.

**Keywords:** cellular-level technology, EndoScell, frozen section, papillary thyroid carcinoma, thyroid nodules

## Abstract

**Rationale:**

Accurate resection of papillary thyroid carcinoma (PTC) is key to prognosis, but frozen section has sampling and time limitations.

**Patient concerns:**

A 50-year-old female was diagnosed with suspected PTC and underwent surgical treatment.

**Diagnoses:**

Intraoperative EndoCell detected residual atypical cells, and postoperative pathology confirmed bilateral PTC.

**Interventions:**

The patient underwent a total thyroidectomy, with intraoperative EndoCell (real-time cellular imaging) used with nanocarbon/indocyanine green to assess margins and preserve parathyroids.

**Outcomes:**

EndoCell guided complete resection of residual tumor, with parathyroids preserved and no severe postoperative complications.

**Lessons:**

EndoCell overcomes the limitations of frozen section, enhancing PTC surgical precision and safety when combined with complementary imaging.

## 1. Introduction

Thyroid cancer is one of the most prevalent malignant tumors worldwide, with its incidence showing a steady upward trend globally.^[1]^ Among all subtypes of thyroid cancer, papillary thyroid carcinoma (PTC) is the most common pathological type in both adults and children, accounting for 80% of all thyroid cancer cases and 85% of differentiated thyroid cancer cases.^[2]^ It predominantly affects females aged 30 to 50 years, featuring well-differentiated morphology and low malignant potential. Despite the high incidence of multifocal lesions and early cervical lymph node metastasis, PTC generally confers a favorable prognosis.

Cervical ultrasound serves as a key auxiliary examination for thyroid lesions, while fine-needle aspiration (FNA) cytology is an important preoperative diagnostic method for thyroid nodules or suspicious lymph nodes. Clinical suspicion of thyroid cancer should be raised if the thyroid mass is hard and fixed, accompanied by cervical lymphadenopathy, or if a long-standing thyroid nodule exhibits rapid enlargement in a short period.

During surgery, frozen section (FS) analysis is currently the most reliable and practical method for determining resection margins, and intraoperative FS has become an essential tool for confirming diagnosis and guiding appropriate surgical planning.^[3]^

However, FS has several inherent limitations. It primarily serves to distinguish benign from malignant tissue in suspicious regions, but its diagnostic performance is constrained by sampling bias, dependence on experienced pathologists, and time-consuming procedures. These issues become particularly evident when the tumor invades adjacent vital structures such as the trachea. As a result, surgical decision-making is frequently based on intraoperative visual judgment and experience rather than definitive pathological evidence.

To address these challenges, cell-level imaging technologies have been introduced in surgical oncology, offering direct visual guidance for margin assessment in cancers such as gliomas, breast tumors, and thyroid carcinomas. These approaches have significantly improved intraoperative precision and safety.^[4]^ The EndoScell system represents a next-generation cellular imaging device, employing advanced optics and image processing to achieve real-time, in vivo visualization at the cellular level. This enables more accurate determination of tumor boundaries and residual disease, supporting maximal safe resection.

Given the clinical dilemmas of FS limitation and parathyroid protection difficulty in PTC surgery, this study aims to clarify the core value of EndoScell cellular-level real-time imaging technology in intraoperative application for PTC. The specific objectives include: Verifying the accuracy of EndoScell in intraoperative tumor margin assessment and residual lesion identification of PTC, and clarifying whether it can make up for the limitations of traditional FS such as sampling bias and long time consumption; exploring the synergistic application scheme of EndoScell with nanocarbon staining and indocyanine green (ICG) fluorescence imaging, and evaluating its auxiliary role in parathyroid localization and functional protection; preliminarily analyzing the impact of EndoScell on the precision and safety of PTC surgery, so as to provide clinical evidence for optimizing the intraoperative decision-making process of complex PTC (such as cases with tumor invasion of trachea and complex anatomical structures).

In this report, we describe a case in which EndoScell was utilized intraoperatively to assess residual PTC lesions. When used in combination with traditional FS analysis, EndoScell provided rapid and accurate evaluation of surgical margins at the cellular level. Notably, in anatomically complex or deep regions where FS is difficult to implement, EndoScell offered a practical and effective real-time alternative. In addition, real-time exploration of the parathyroid glands (PGs) with EndoScell helped preserve their anatomical and vascular integrity, minimizing the risk of accidental excision and significantly reducing the incidence of postoperative complications.

## 2. Case report

This study has been approved by the Hebei General Hospital Ethics Committee (Approval No. 2025-LW-0306).

A 50-year-old woman with a long-standing history of bilateral thyroid nodules presented with recent nodule enlargement but no symptoms such as pain, hoarseness, dysphagia, or dyspnea. Preoperative ultrasound identified a hypoechoic right lobe nodule (TI-RADS 4a) warranting FNA, which revealed atypical follicular epithelial cells suspicious for papillary thyroid carcinoma (PTC). Additional bilateral nodules were classified as TI-RADS 2 to 3, with bilateral cervical lymphadenopathy.

**Figure 1.**
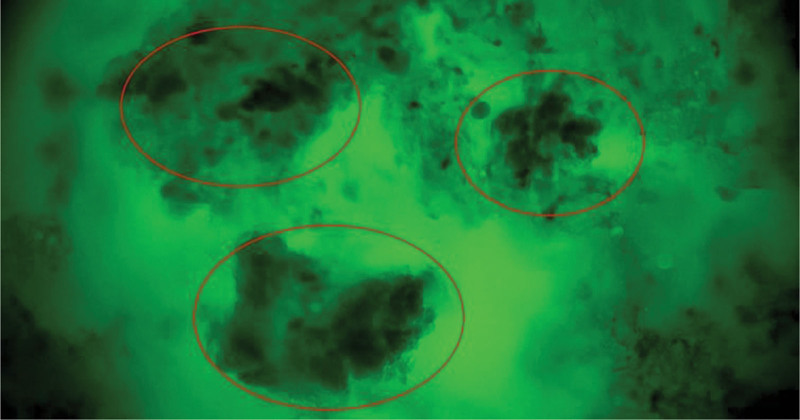
Tumor-infiltrated trachea: the tumor cells form irregular nests, exhibiting markedly enlarged cell bodies and nuclear atypia; tumor cells of varying size are highlighted by red circles, scale bar = 50 μm.

The patient underwent bilateral total thyroidectomy with central neck dissection. Upon exploration, the right thyroid lobe tumor was found tightly adherent to the anterior tracheal wall. The mass measured approximately 0.7 cm × 0.4 cm × 0.6 cm and was firm and solid. The right thyroid lobe and isthmus, along with the pyramidal lobe and prelaryngeal lymph nodes, were carefully dissected and removed. During this step, both nanocarbon staining and ICG fluorescence were helpful in identifying lymph nodes, which were then confirmed intraoperatively to show clear fluorescence and black staining. The superior and inferior parathyroid glands were clearly visualized with combined ICG fluorescence and EndoScell cellular-level imaging, allowing preservation of glandular structure and vascular supply. Immediately after excision of the tumor, EndoScell technology was used to evaluate the exposed anterior tracheal wall. The first round of EndoScell imaging revealed residual atypical cells, prompting additional scraping of the anterior fascia and superficial tracheal cartilage (Fig. 1). A second application of EndoScell confirmed no residual abnormal cells, supporting complete tumor removal.

**Figure 2.**
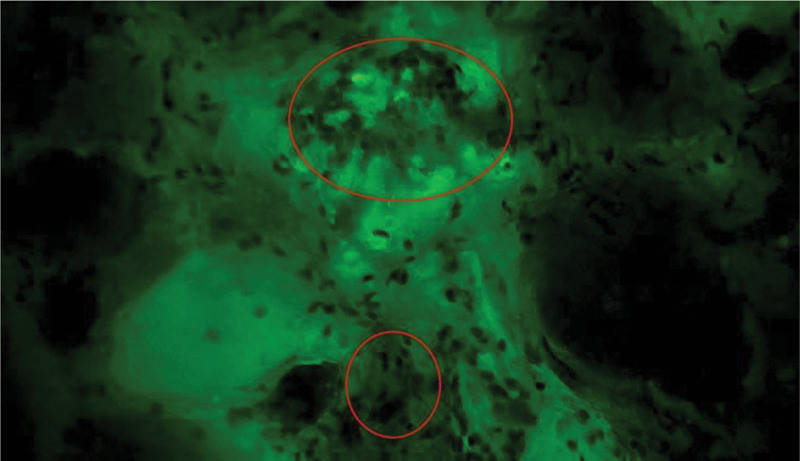
Papillary thyroid carcinoma tissue with papillary structures, disordered cell arrangement, and evident nuclear atypia, scale bar = 50 μm.

The left thyroid lobe was then completely excised using the same approach. Both the left superior and inferior parathyroid glands were successfully identified and preserved. No obvious parathyroid tissue was seen in the resected specimen macroscopically. Frozen section (FS) was performed on the right thyroid tumor, prelaryngeal lymph nodes, and the scraped tracheal wall. EndoScell imaging of the excised tumor confirmed the presence of multiple nests of atypical cells, consistent with malignancy (Fig. 2). FS confirmed papillary carcinoma in the right lobe, no malignancy in the tracheal wall, and no cancer in the prelaryngeal lymph nodes.

The central compartment was then systematically cleared. Several lymph nodes on both sides of level VI showed distinct carbon staining and ICG fluorescence, indicating successful uptake of dual tracers. EndoScell was used intraoperatively to examine suspicious lymph nodes with enhanced signals, assisting in the selection of nodes for frozen pathology. Final FS revealed one metastatic lymph node on the right, while others were benign.

The surgical site was irrigated, checked for bleeding, and a drain was placed before layered closure.

The final paraffin pathology report confirmed bilateral papillary thyroid carcinoma: a dominant lesion in the right lobe and a 0.2 cm microcarcinoma in the left lobe, with no malignancy in the tracheal tissue. No parathyroid glands were excised, and the patient remained normocalcemic postoperatively.

The patient recovered well postoperatively, with no complaints of choking, palpitations, or chest discomfort. She experienced transient dysphagia and mild fatigue, both of which resolved without treatment.

All personal clinical data of the reported patient, including preoperative examination results (ultrasound, FNA cytology), intraoperative surgical records, pathological reports (FS and paraffin section), and postoperative follow-up data, are properly archived in the electronic medical record system of Hebei Provincial People’s Hospital (Shijiazhuang, China).

## 3. Discussion

Papillary thyroid carcinoma (PTC) is the most common type of thyroid cancer. Clinical practice guidelines consistently emphasize that surgery is the core treatment modality for thyroid cancer. For differentiated thyroid carcinoma (DTC), postoperative ^131^I therapy and thyroid-stimulating hormone (TSH) suppression therapy are important adjuvant treatments; in contrast, systemic therapies (such as radiotherapy and targeted therapy) play a crucial role in the management of anaplastic thyroid carcinoma (ATC), advanced medullary thyroid carcinoma, and refractory DTC.^[5]^ Application of EndoSCell enabled direct visualization of pleomorphic cell clusters characteristic of PTC, including papillary structures with central fibrovascular cores and psammoma bodies.^[6]^ Notably, EndoSCell was used intraoperatively to examine the resection margin at the anterior tracheal wall, which showed no residual atypical cells following tumor dissection. This allowed the surgical team to avoid excessive tissue removal while confidently achieving negative margins – an advantage over conventional FS pathology in anatomically constrained regions.

Prevention of postoperative hypoparathyroidism is another critical goal during total thyroidectomy. In this context, ICG fluorescence enabled intraoperative identification of PGs and their vasculature, while EndoSCell provided immediate confirmation of parathyroid cellular morphology and viability. This dual approach reduced the risk of inadvertent PG injury and postoperative hypocalcemia.

Previous studies have demonstrated that EndoSCell achieves over 90% concordance with hematoxylin-eosin-stained histopathology and offers comparable diagnostic accuracy to FS, but with substantially shorter processing times.^[4]^ This makes it an ideal tool for high-stakes intraoperative decision-making where speed and precision are essential.

In conclusion, this case highlights the clinical value of EndoSCell for real-time microscopic assessment during PTC surgery. When combined with complementary technologies such as nanocarbon and ICG fluorescence imaging, EndoSCell enhances surgical navigation, improves tumor margin evaluation, and supports preservation of critical structures including the parathyroid glands. These findings support its integration into a multimodal intraoperative workflow alongside FS pathology, offering significant advantages in surgical safety, efficacy, and oncological outcomes.

In the surgical practice of thyroid cancer, the significance of this study lies in: on the one hand, aiming at the clinical pain point that FS is limited in the assessment of key sites (such as paratracheal region), it provides a real-time and accurate cellular-level imaging solution, helping to achieve the surgical goal of “maximizing tumor resection + minimizing normal tissue damage”; on the other hand, through the exploration of multi-technology collaborative mode, it provides a new technical idea for reducing the incidence of postoperative complications such as hypoparathyroidism, further improves the intraoperative precise navigation system of PTC, and promotes the development of thyroid surgery towards “individualization, minimally invasiveness and precision.”

## 4. Future perspectives

Integrating multimodal imaging technologies will further expand the application scope of EndoScell. This study combines EndoScell with nanocarbon staining and indocyanine green (ICG) fluorescence imaging, but the integration is still at the level of parallel application. Future development can explore the organic fusion of EndoScell with molecular imaging, ultrasound, or computed tomography (CT) technologies. For example, labeling specific molecular markers of PTC (such as BRAF V600E) with fluorescent probes that can be recognized by EndoScell will enable targeted imaging of tumor cells, further improving the specificity of residual lesion detection. In addition, real-time fusion of EndoScell’s cellular-level images with intraoperative ultrasound or CT’s anatomical images can help surgeons more intuitively correlate microscopic cellular changes with macroscopic anatomical structures, enhancing surgical decision-making accuracy.

## Author contributions

**Conceptualization:** Zhuoyang Zhou, Lixiao Ma.

**Supervision:** Zeyu Zhang, Fenghua Zhang.

**Writing – original draft:** Yaqian Liu.
